# Degradation of silicon photonic biosensors in cell culture media: analysis and prevention

**DOI:** 10.1364/BOE.8.002924

**Published:** 2017-05-09

**Authors:** Graham J. Triggs, Gareth J. O. Evans, Thomas F. Krauss

**Affiliations:** 1 Department of Physics, University of York, York YO10 5DD, UK; 2 Department of Biology, University of York, York YO10 5DD, UK

**Keywords:** (280.4788) Optical sensing and sensors, (120.5700) Reflection, (350.1820) Damage, (310.1515) Protective coatings

## Abstract

Silicon photonic biosensors are being widely researched as they combine high performance with the potential for low-cost mass-manufacturing. Sensing is typically performed in an aqueous environment and it is assumed that the sensor is chemically stable, as silicon is known to etch in strong alkaline solutions but not in liquids with a pH close to 7. Here, we show that silicon can be affected surprisingly strongly by typical cell culture media, and we observe etch rates of up to 2 nm/hour. We then demonstrate that a very thin (< 10 nm) layer of thermal oxide is sufficient to suppress the etching process and provide the long-term stability required for monitoring cells and related biological processes over extended periods of time. We also show that employing an additional pH buffering compound in the culture medium can significantly reduce the etch rate.

## 1. Introduction

Silicon photonics has become a firmly established technology, both in the fields of data communications and in biomedical sensing. The sensing application relies on resonant nanostructures such as microrings and photonic crystals. Microring resonators have demonstrated limits of detection down to 10^−7^ RIU (refractive index units) [1], and they have seen commercial success as a healthcare diagnostic tool [2]. Photonic crystals [3–5] can detect molecular monolayers with masses in the low femtogram range [5].

Despite the extensive use of silicon in optical biosensors, to our knowledge, there is no study on its stability in standard cell culture medium (CCM). CCM is one of the most commonly used environments in biological and medical research. Moreover, with the development of multiple sensors arrayed on a single chip [6], combined electro-photonic sensors [7], and fibre-tip sensors that can be inserted into cells [8], biosensing *in vitro* or even *in vivo* is becoming a realistic possibility, and the stability of silicon in a live cell environment is becoming an important question. Stability is therefore not only a concern for the integrity of the photonic structures, but also for the viability of cells in the presence of silicon-based byproducts produced upon degradation.

**Fig. 1:**
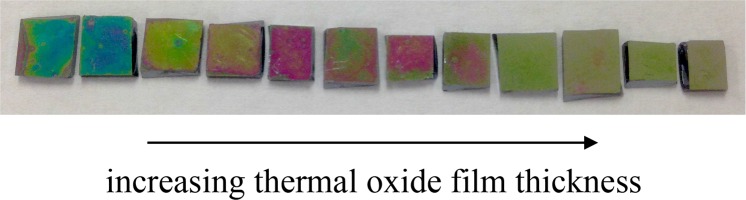
Photograph of SOI samples after being incubated in CCM for 96 hours. The samples have been treated with a thermal oxide film for protection, with oxide thickness increasing from left to right. The samples on the left have been etched significantly and changed their colour, while the samples on the right are untouched and retain their original colours.

In this paper, we demonstrate the drastic effect of the CCM on silicon: following a typical cell culture period of 4 days, up to 85% of the 220 nm thick device layer on silcon-on-insulator (SOI) is etched away (see fig. 1). This degree of etching clearly makes a waveguide-based optical sensor unuseable; moreover, even the very small thickness change incurred over a few hours would already be noticeable, especially given the extreme sensitivities of these sensors to refractive index changes. After analysing the etching of silicon by the CCM, we first employ a simple solution to prevent the etching by passivating the silicon with a thin (< 10 nm) thermal oxide layer (see fig. 1). Silicon oxide has previously been reported as a passivation layer for silicon devices in aqueous environments [9, 10]. Secondly, we show that the SOI etching rate can be greatly reduced by improving the pH buffering of the CCM.

## 2. Materials and methods

### 2.1. Materials used

The optical material used here is silicon-on-insulator (SOI, Soitec, France), with a nominal device layer thickness (*t_dev_*) of 220 nm and a buried oxide layer thickness of ∼ 2000 nm. This value of *t_dev_* is commonly used in the community, being designed to support single-mode operation at 1550 nm wavelength. All SOI samples were cleaved from the same section of a wafer to minimise variation between samples, and were cleaned in acetone and isopropanol alcohol in an ultrasonic bath.

For the degradation tests, we used two of the most common types of CCM: DMEM (Dulbecco’s modified Eagle medium, Thermo Fisher Scientific, 41966029), which we refer to as “medium A” and RPMI 1640 (Roswell Park Memorial Institute medium, Thermo Fisher Scientific, 31870025), “medium B”. These are basal media containing nutrients, salts, hormones and growth factors to promote cell viability. Like many CCMs, they have a sodium bicarbonate pH buffer system, designed for use in an elevated CO_2_ atmosphere. The bicarbonate ions balance dissolved CO_2_ from the atmosphere to maintain a pH of 7.4 at 5–10% CO_2_ [11]. All samples were sterilised in a 70% ethanol:water solution before being added to petri dishes containing CCM and placed in a cell culture incubator at 37°C and 5% CO_2_.

### 2.2. Thermal oxidation of SOI

For the thermal oxidation of SOI, we used a barrel furnace (Carbolite, MTF 12/50/400) with a dry atmosphere and a flow of 0.5 sccm of oxygen. The oxidation time was fixed at 10 minutes for all samples, while the furnace temperature was varied to control the oxide thickness. Control samples were retained that did not undergo thermal oxidation. The native oxide on the SOI was not removed from any of the samples.

### 2.3. Determination of the SOI device layer thickness (*t_dev_*)

We employ reflectometry to determine the device layer thickness, *t_dev_*. Given an accurate knowledge of the complex refractive index of the materials involved (Si and SiO_2_), a measured reflectance spectrum is readily compared to a lookup table containing simulated reflectance spectra with different *t_dev_*. For the simulations, we employed rigorous coupled-wave analysis (RCWA) [12], implemented in MATLAB. Refractive index data for Si were obtained from [13], while for SiO_2_ it was set to 1.47, as it varies only slightly over our wavelength range of interest, and is in good agreement with typical values found in the literature. For the measurements, we used a simple reflectometer setup, which enables the reflectance spectrum to be obtained from a ∼ 100 *μm* spot on the sample with a Thorlabs CCS175 spectrometer. Due to non-uniform etching by the CCM, reflectance spectra were measured at multiple locations across each sample.

**Fig. 2:**
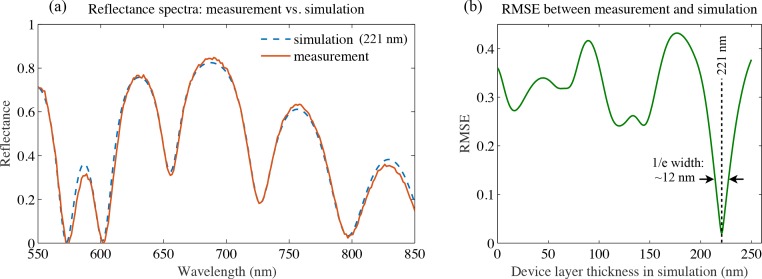
(a) Comparison between a measured reflectance spectrum (orange) and a simulation (blue) of SOI with *t_dev_* = 221 nm. (b) Plot of the RMSE between the measured curve in (a) and the simulation for different values of *t_dev_*. The best match corresponds to *t_dev_* = 221 nm, where the RMSE is minimised. The 1/e width of the curve is measured to be 12 nm, and this is used as a systematic error in determining *t_dev_*.

After measuring a reflectance spectrum, it is matched to a simulated reflectance spectrum by identifying the value of *t_dev_* that minimises the root-mean-square error (RMSE) between the two curves. We used data in the wavelength range 550–850 nm. Figure 2(a) shows a measured spectrum (orange) with the best-matching simulated curve (blue dashes). Figure 2(b) shows the RMSE between the measurement and the simulation for a range of values of *t_dev_*. The RMSE is clearly minimised at *t_dev_* = 221 nm, in excellent agreement with the quoted value of 220nm.

### 2.4. Measurement of the thermal oxide thickness

Determining the thickness of the thermally-grown silicon oxide films was difficult due to the low oxide thicknesses studied here. This, combined with the low refractive index of silica, meant reflectometry measurements were not reliable. Therefore, we employed ellipsometry to measure the oxide thickness.

## 3. Results

### 3.1. Analysis of SOI etching by cell media

**Fig. 3:**
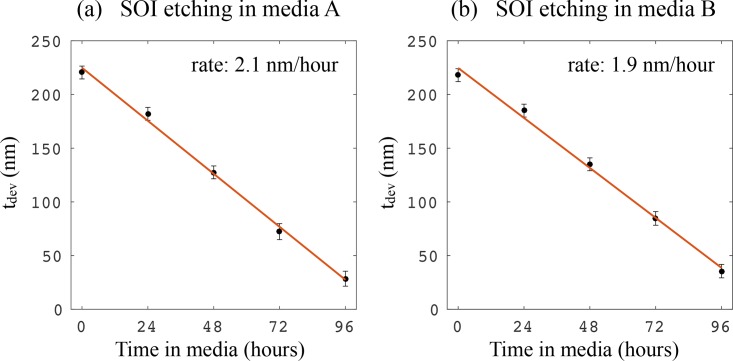
*t_dev_* (remaining device layer thickness) against time in (a) media A (DMEM) and (b) media B (RPMI 1640). The slopes of the fitted straight lines (orange) provide the etching rates.

Figures 3(a) and 3(b) show *t_dev_* versus time spent in the two different media. In both cases, the thickness linearly decreases with time, for a duration of up to 4 days. Strikingly, after 96 hours, as much as ∼ 85% of the device layer has been etched away. The slopes of fitted straight lines are used to deduce etching rates of 2.1 nm/hour and 1.9 nm/hour for media A and B, respectively.

### 3.2. Prevention of SOI etching with a thin thermal oxide layer

**Fig. 4:**
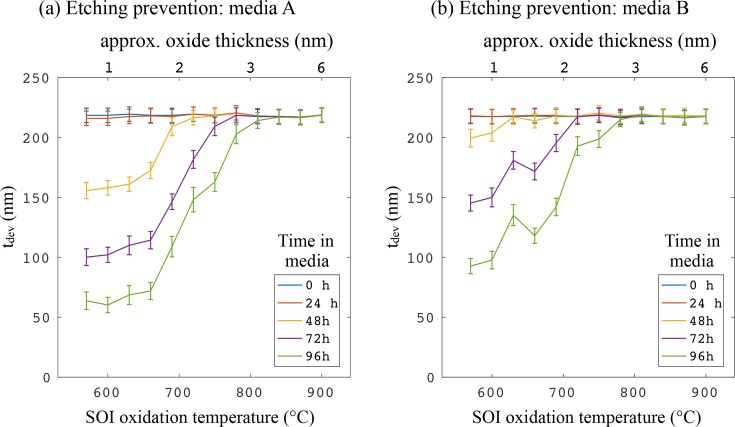
*t_dev_* (remaining device layer thickness) against thermal oxidation temperature for different times spent in (a) media A (DMEM) and (b) media B (RPMI 1640). Shown along the top x-axis is the approximate oxide thickness.

Next, we present a method for preventing the etching of SOI by CCM by passivating the top surface with a thermally-grown oxide film. To study the role of oxide thickness, 12 samples were oxidised at a range of temperatures from 570°C to 900°C in increments of 30°C. Following oxidation, *t_dev_* (the remaining device layer thickness) was measured for each sample after spending 0, 24, 48, 72 and 96 hours in both types of media. Figure 4 shows the resulting measurements, where the error bars are the 12 nm systematic error discussed above, plus the standard deviation of multiple measurements at different locations on each sample (to take into account uneven etching). Furthermore, the approximate oxide thickness resulting from oxidation at these temperatures is shown along the upper horizontal axes. These values have been determined from an oxide growth model and ellipsometry data, as discussed later in this paper.

**Fig. 5:**
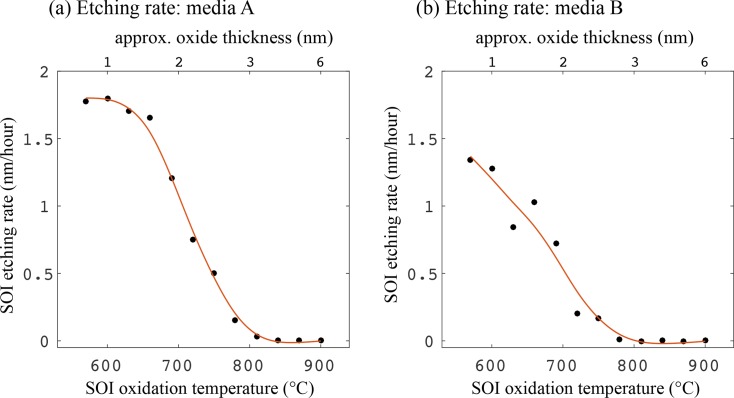
Device layer etching rate over 96 hours, against thermal oxidation temperature for SOI in (a) media A (DMEM) and (b) media B (RPMI 1640). Shown along the top x-axis is the approximate oxide thickness. This data is obtained from that shown in Fig. 4, and the red lines are smoothed versions of the data.

It is evident that there is only a minimal change after 24 hours (orange curves), for both media types. After this, however, the samples with the thinnest oxide film (i.e. lower oxidation temperature) began to be etched. As the time spent in the media increases, a thicker oxide layer is required to protect the SOI, and after 94 hours, only those samples oxidised at 840°C and above remain at full thickness. The etching action is similar, but slightly slower, for medium B, consistent with the slightly lower etch rate found in fig. 3. Both graphs show a gradual transition between etching and protection, depending on the oxide thickness (oxidation temperature). Using the data from Fig. 4, the average etch rates over 96 hours are calculated for each oxidation temperature, and this is shown in Fig. 5. It is clearly seen that the oxidation reduces the etching rate to zero for both types of media.

### 3.3. Estimation of the thermal oxide film thickness

**Fig. 6:**
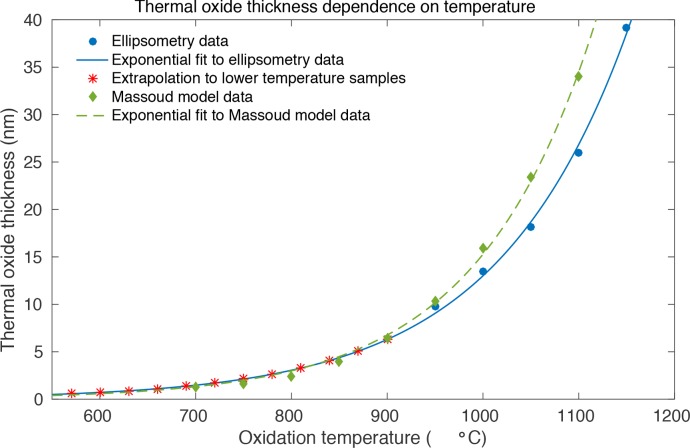
Ellipsometry measurements of oxide films on SOI (blue dots). An exponential curve (blue line) is fitted to this data and used to estimate the thicknesses of the oxide films at lower temperatures, as used in the CCM etching experiments (red stars). The green diamonds show data obtained from [16], which uses the Massoud model of oxide growth, while the green dashed line is an exponential fit. We note the difference at higher temperatures may be due to a small error in furnace operation temperature of 20–30 °C.

Having proven that thermal oxidation is a viable method to prevent SOI from being etched in CCM, it is important to consider the thickness of the resulting thermal oxide layer as this will impact on the optical properties of a photonic device made from the oxidised silicon. We note that the oxide layer may even be beneficial for the photonic performance, as thermal oxidation of SOI waveguides has been shown to reduce surface roughness and related propagation losses [14]. We estimate that the oxide films required for complete passivation (>840°C) are only thin (<10 nm), according to the Massoud model of thermal oxide growth [15, 16]. The relatively low oxidation temperature, dry oxidising atmosphere, and short oxidation time of just 10 minutes substantiate this. Furthermore, we observe no apparent difference between the reflected colour of the various SOI samples after oxidation, which is a surprisingly sensitive way to estimate SOI oxide thickness [17]. We employed ellipsometry to determine the oxide thickness for various samples oxidised at different temperatures. Because the oxide thickness is so low, it was difficult to make accurate measurements, so additional SOI samples were oxidised at even higher temperatures than used for the etching measurements. The resulting thicker oxide films can be measured more reliably (see fig. 6), and the thickness can then be extrapolated down using the well-known exponential relationship [16,18]. Our results suggest that the minimum oxide layer thickness required to protect SOI for 96h in media A is approximately 4 nm, achieved with an oxidation temperature of 840°C.

### 3.4. Reducing the etching rate of SOI using buffered CCM

**Fig. 7:**
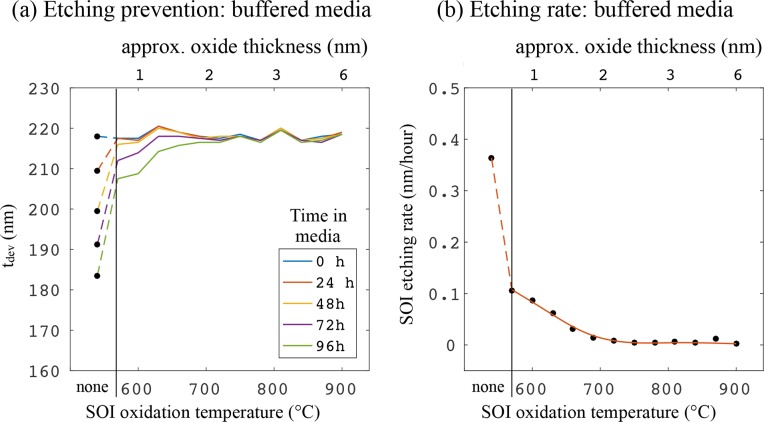
(a) *t_dev_* (remaining device layer thickness) against thermal oxidation temperature for different times spent in buffered CCM. Shown along the top x-axis is the approximate oxide thickness. Note the significantly reduced etching compared to non-buffered CCM in fig. 4, evident from the re-scaled y-axis. (b) Device layer etching rate against thermal oxidation temperature for SOI in buffered CCM. The dashed sections of these curves join to the data points from samples that received no oxidation treatment, for comparison. Error bars are omitted from (a) to more clearly show the data; indeed, the etching by buffered CCM is almost as low as the uncertainty in thickness measurement.

Next, we present a method for significantly reducing the etching rate of SOI in CCM by including an additional pH buffering agent in the medium. It is common to use an additional buffer when the culture is to be removed from an elevated CO_2_ atmosphere for a prolonged period, for example during time-lapse imaging. HEPES (4-(2-hydroxyethyl)-1-piperazineethanesulfonic acid) is often used in this case, and it is known that it supplements the pH buffering provided by the standard bicarbonate system in the pH range 7.2–7.6 [19]. HEPES is an organic compound that does not require an elevated CO_2_ atmosphere to maintain the correct pH of the CCM. Here, we use HEPES-buffered DMEM (Thermo Fisher Scientific, 21063029). Figure 7(a) shows the measurements of *t_dev_* for each sample after spending 0, 24, 48, 72 and 96 hours in the buffered CCM. Included are points from samples that received no thermal oxidation. For all oxidised samples, the etching rate is 0.1 nm/hour or slower: significantly lower than for non-buffered CCM. Even non-oxidised SOI is etched quite slowly in the buffered CCM (∼ 0.37 nm/hour).

## 4. Discussion and conclusions

It is well known that crystalline silicon is chemically etched by alkaline solutions, and this is often used in silicon processing to etch away silicon. Common wet etchants of silicon are hydroxide solutions, namely potassium hydroxide (KOH) and tetramethylammonium hydroxide (TMAH), typically used at pH >12. We suggest that the etching of SOI by CCM, as reported here, is also caused by the slight alkalinity of the solution (nominal CCM pH is 7.4 for mammalian cells). In particular, there is a high concentration of sodium bicarbonate in DMEM (3.7 g/L), and this has been reported to etch crystalline silicon on its own [20]. In basic solutions, hydroxide ions (OH^−^) initiate the hydrolysis reaction: $\mathrm{Si}+2{\mathrm{OH}}^{-}+2H_{2}O\to {\mathrm{SiO}}_{2}{\left(\mathrm{OH}\right)}_{2}^{2-}+2H_{2}$ [21]. The significant reduction in etch rate observed when using the HEPES-buffered CCM may be due to the superior control of the pH of the solution over extended periods of time, where it is prevented from becoming too alkaline.

Our result, while somewhat surprising, is not altogether unexpected. Silicon nanomembranes have previously been studied as biocompatible electronic systems that can dissolve after a prescribed time, e.g. for implantable medical devices [22, 23] and transient electronics [24]. In [22], the dissolution of the silicon occurred at a rate of 4.5 nm/day in PBS at pH 7.4 at 37 °C. This etch rate is substantially slower than the one we observe for non-oxidised silicon (∼ 50 nm/day, fig. 3), which suggests that the (many) other ingredients present in the CCM may contribute to the etch rate. Although the duration of our experiments (4 days) is representative of completing many cell-based assays, the question of long-term durability remains. This is particularly applicable to sensor chips that can be re-used for multiple experiments, and as such, further studies into the stability of oxide-passivated silicon over longer periods of time would be beneficial. Cell viability and cell adhesion on crystalline silicon and silicon carbide (SiC) surfaces has been investigated, and it was found that SiC provides a more biocompatible substrate for cell culture than silicon [25]. Silicon nitride has also been reported as an effective silicon passivation material [9,26], and has been used as a surface on which to culture cells [27,28].

We have analysed the stability of SOI in two different types of cell culture media by measuring the thickness of the device layer using reflectometry. After 96 hours incubation, up to 85% of the device layer has been etched away. By thermally oxidising the SOI at temperatures above 840°C, a thin (<10 nm) oxide layer is grown, which prevents etching. Furthermore, we show that by adding an additional pH buffering compound to the CCM, the etch rate can be greatly reduced.

## References

[1] Iqbal M. Gleeson M. A. Spaugh B. Tybor F. Gunn W. G. Hochberg M. Baehr-jones T. Bailey R. C. Gunn L. C., “Label-Free Biosensor Arrays Based on Silicon Ring Resonators and High-Speed Optical Scanning Instrumentation,” IEEE J. Sel. Top. Quantum Electron. 16, 654–661 (2010). 10.1109/JSTQE.2009.2032510

[2] Genalyte, “Maverick Detection System,” URL: www.genalyte.com/about-us/our-technology/, accessed 2016-12-05.

[3] Scullion M. G. Di Falco A. Krauss T. F., “Biosensors and Bioelectronics Slotted photonic crystal cavities with integrated microfluidics for biosensing applications,” Biosens. Bioelectron. 27, 101–105 (2011). 10.1016/j.bios.2011.06.023 21764290

[4] Mandal S. Goddard J. M. Erickson D., “A multiplexed optofluidic biomolecular sensor for low mass detection,” Lab Chip 9, 2924–2932 (2009). 10.1039/b907826f 19789745

[5] Pal S. Guillermain E. Sriram R. Miller B. L. Fauchet P. M., “Silicon photonic crystal nanocavity-coupled waveguides for error-corrected optical biosensing,” Biosens. Bioelectron. 26, 4024–4031 (2011). 10.1016/j.bios.2011.03.024 21524903 PMC3104068

[6] Scullion M. G. Fischer M. Krauss T. F., “Fibre Coupled Photonic Crystal Cavity Arrays on Transparent Substrates for Spatially Resolved Sensing,” Photonics 1, 412–420 (2014). 10.3390/photonics1040412

[7] Juan-Colás J. Parkin A. Dunn K. E. Scullion M. G. Krauss T. F. Johnson S. D., “The electrophotonic silicon biosensor,” Nat. Commun. 7, 12769 (2016). 10.1038/ncomms12769 27624590 PMC5027286

[8] Shambat G. Kothapalli S.-R. Provine J. Sarmiento T. Harris J. Gambhir S. S. Vučković J., “Single-Cell Photonic Nanocavity Probes,” Nano Lett. 13, 4999–5005 (2013). 10.1021/nl304602d 23387382 PMC3686990

[9] Seidel H. Csepregi L. Heuberger A. Baumgärtel H., “Anisotropic etching of crystalline silicon in alkaline solutions i. orientation dependence and behavior of passivation layers,” J. Electrochem. Soc. 137, 3612–3626 (1990). 10.1149/1.2086277

[10] Cattarin S. Musiani M. M., “Electrodissolution and passivation of silicon in aqueous alkaline media: A voltammetric and impedance investigation,” J. Phys. Chem. B 103, 3162–3169 (1999). 10.1021/jp982462t

[11] ThermoFisher Scientific, “pH & CO2 levels,” URL: www.thermofisher.com/uk/en/home/references/gibco-cell-culture-basics/cell-culture-environment/ph-co2-levels.html, accessed 2016-12-05.

[12] Moharam M. G. Grann E. B. Pommet D. A. Gaylord T. K., “Formulation for stable and efficient implementation of the rigorous coupled-wave analysis of binary gratings,” J. Opt. Soc. Am. A 12, 1068–1076 (1995). 10.1364/JOSAA.12.001068

[13] Palik E. D., Handbook of Optical Constants of Solids (Academic Press, Boston, 1985).

[14] Pafchek R. Tummidi R. Li J. Webster M. A. Chen E. Koch T. L., “Low-loss silicon-on-insulator shallow-ridge TE and TM waveguides formed using thermal oxidation,” Appl. Opt. 48, 958–963 (2009). 10.1364/AO.48.000958 19209210

[15] Massoud H. Z. Plummer J. D., “Analytical relationship for the oxidation of silicon in dry oxygen in the thin - film regime,” J. Appl. Phys. 62, 3416–3423 (1987). 10.1063/1.339305

[16] Perozziello E., “Silicon Thermal Oxide Thickness Calculator,” URL: www.lelandstanfordjunior.com/thermaloxide.html, accessed 2016-12-02 (2015).

[17] Henrie J. Kellis S. Schultz S. M. Hawkins A., “Electronic color charts for dielectric films on silicon,” Opt. Express 12, 1464–1469 (2004). 10.1364/OPEX.12.001464 19474970

[18] Filipovic L., “2.2.1.3 Kinetics and Growth of Silicon Dioxide: Temperature Effects,” URL: www.iue.tuwien.ac.at/phd/filipovic/node29.html.

[19] ThermoFisher Scientific, “HEPES,” URL: www.thermofisher.com/uk/en/home/life-science/cell-culture/mammalian-cell-culture/reagents/hepes.html, accessed 2016-12-02.

[20] Ma J.-j. Luo R.-z. Wang Y.-q. Man S.-q., “Microstructure Fabricated by Monocrystalline Silicon Anisotropic Etching in Sodium Carbonate and Sodium Bicarbonate Solutions,” in “Int. Conf. Electromechanical Control Technol. Transp.”, (2015), pp. 570–575.

[21] Gonzalez-Pereyra N. G., “Anisotropic etching of monocrystalline silicon under subcritical conditions,” Ph.D. thesis, All Dissertations, Paper 1497 (2015).

[22] Hwang S.-W. Tao H. Kim D.-H. Cheng H. Song J.-K. Rill E. Brenckle M. A. Panilaitis B. Won S. M. Kim Y.-S. Song Y. M. Yu K. J. Ameen A. Li R. Su Y. Yang M. Kaplan D. L. Zakin M. R. Slepian M. J. Huang Y. Omenetto F. G. Rogers J. A., “A Physically Transient Form of Silicon Electronics,” Science (80-.). 337, 1640–1644 (2012). 10.1126/science.1226325 23019646 PMC3786576

[23] Yin L. Farimani A. B. Min K. Vishal N. Lam J. Lee Y. K. Aluru N. R. Rogers J. A., “Mechanisms for hydrolysis of silicon nanomembranes as used in bioresorbable electronics,” Advanced Materials 27, 1857–1864 (2015). 10.1002/adma.201404579 25626856

[24] Kang S.-K. Park G. Kim K. Hwang S.-W. Cheng H. Shin J. Chung S. Kim M. Yin L. Lee J. C. Lee K. M., “Dissolution chemistry and biocompatibility of silicon-and germanium-based semiconductors for transient electronics,” ACS Appl. Mater. Interfaces 7, 9297–9305 (2015). 10.1021/acsami.5b02526 25867894

[25] Coletti C. Jaroszeski M. J. Pallaoro A. Hoff A. M. Iannotta S. Saddow S. E., “Biocompatibility and wettability of crystalline SiC and Si surfaces,” in “Proc. 29th Annu. Int. Conf. IEEE EMBS,” (2007), 8, pp. 5849–5852. 10.1109/IEMBS.2007.4353678 18003344

[26] Regonda S. Tian R. Gao J. Greene S. Ding J. Hu W., “Silicon multi-nanochannel fets to improve device uniformity/stability and femtomolar detection of insulin in serum,” Biosens. Bioelectron. 45, 245–251 (2013). 10.1016/j.bios.2013.01.027 23500371

[27] Dhakal A. Wuytens P. Peyskens F. Skirtach A. Le Thomas N. Baets R., “Microscope-less lab-on-a-chip raman spectroscopy of cell-membranes,” in “Photonics Conference (IPC), 2016 IEEE,” (IEEE, 2016), pp. 144–145.

[28] Triggs G. J., “Resonant grating surfaces for biosensing,” Ph.D. thesis, University of York (2016).

